# Erythema multiforme-like drug eruption in a patient with mycosis fungoides on durvalumab and lenalidomide

**DOI:** 10.1016/j.jdcr.2026.07.037

**Published:** 2026-07-29

**Authors:** Liliana L. Crisan, Jeralyn Quach, Jasmine Zain, Steven T. Rosen, Christiane Querfeld

**Affiliations:** a Division of Dermatology, City of Hope National Medical Center and Beckman Research Institute, Duarte, California; b Department of Pathology, City of Hope National Medical Center and Beckman Research Institute, Duarte, California; c Clinical Trials Office, City of Hope National Medical Center and Beckman Research Institute, Duarte, California; d Department of Hematology and Hematopoietic Cell Therapy, City of Hope National Medical Center and Beckman Research Institute, Duarte, California

**Keywords:** CTCL, cutaneous T-cell lymphoma, durvalumab, EM, erythema multiforme, ICIs, immune checkpoint inhibitors, immune-related adverse events, irAEs, lenalidomide, MF, mycosis fungoides, PD-L1, programmed cell death-ligand 1

## Introduction

Immune checkpoint inhibitors (ICIs) have advanced oncologic therapy by blocking inhibitory pathways that restrain immune activation, hereby enabling the immune system to recognize and eliminate malignant cells. Durvalumab is a fully human monoclonal antibody targeting programmed death-ligand 1 (PD-L1), approved by the US Food and Drug Administration (FDA) for the treatment of lung, hepatocellular, biliary tract, endometrial, bladder and gastric cancers.^1^ Recent preclinical and clinical evidence suggests that PD1/PD-L1 blockade may also provide meaningful clinical benefit in patients with recurrent/refractory cutaneous T-cell lymphoma (CTCL).^2^, ^3^, ^4^ Lenalidomide, an immunomodulatory drug approved for multiple hematological malignancies and transfusion-dependent anemia associated with a deletion 5q, has demonstrated single-agent activity in CTCL.^5^ The combination of lenalidomide with durvalumab is hypothesized to have synergistic effects, potentially enhancing anti-tumor immunity by promoting cytotoxic responses and reducing immunosuppression. Despite their promising efficacy, ICIs can trigger a unique spectrum of immune-related adverse events (irAEs), which are thought to result from the disruption of immune tolerance. These toxicities can affect any organ system, with cutaneous irAEs, particularly maculopapular rash and lichenoid dermatitis, among the most frequently observed.^6^^,^^7^ While often mild, these reactions can occasionally be severe, leading to treatment interruption or discontinuation, and pose diagnostic challenges in distinguishing drug toxicity from disease progression. Here we report a case of delayed-onset erythema multiforme-like (EM) drug reaction in a patient treated with durvalumab combined with lenalidomide for mycosis fungoides (MF), the most common type of CTCL.

## Case report

A 69-year-old Caucasian man presented to our multidisciplinary cutaneous lymphoma clinic with disseminated erythematous patches and plaques. Thirteen months prior, he had been diagnosed with MF, clinical stage IB with large cell transformation (LCT). Upon reevaluation, comprehensive staging was performed, including complete blood count (CBC), lactate dehydrogenase, peripheral blood flow cytometry for circulating Sezary cells, 2 skin biopsies for histopathologic evaluation with immunophenotyping (Fig 1, *A*-*F*), T-cell receptor sequencing analysis on skin and blood, and whole-body positron emission tomography/computed tomography scans. These confirmed the diagnosis of MF, stage IB (T2bN0M0B0a) with LCT.

**Fig 1:**
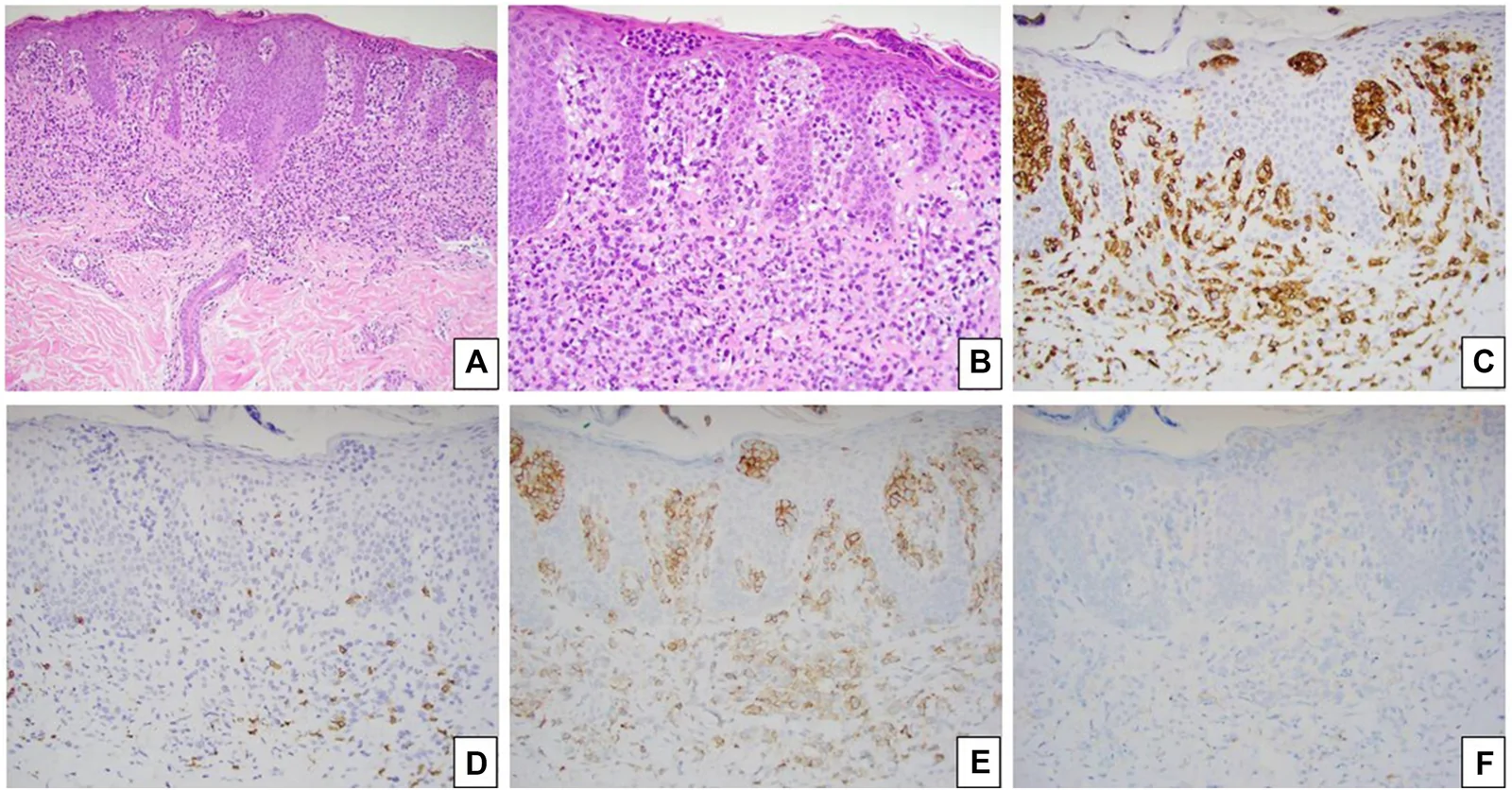
Histopathologic findings of mycosis fungoides show a band-like atypical lymphoid infiltrate composed of atypical lymphocytes, scattered small lymphocytes and histiocytes. There is significant epidermotropism of single atypical lymphocytes and few Pautrier microcollections noted **(A** and **B)** [Hematoxylin-Eosin stain, ×100 and ×200 magnification]. Immunohistochemistry reveals high expression of CD4 **(C)**, negative CD8 **(D)**, high expression of PD1 **(E)**, and very low expression of PD-L1 in histiocytes within the infiltrate **(F)** [×200 magnification].

Initial management included brentuximab vedotin, followed by weekly oral methotrexate and topical triamcinolone cream. After multidisciplinary discussion of treatment options, the patient was enrolled in an investigational protocol evaluating durvalumab with or without lenalidomide and randomized to the combination arm. Topical triamcinolone cream was allowed and continued daily.

At baseline, his skin burden was quantified by a modified severity-weighted assessment tool (mSWAT) score of 67. The treatment included durvalumab (1500 mg IV on day 1 of a 28-day cycle) and oral lenalidomide administered on days 1-21 of each 28-day cycle (10 mg in cycle 1, 15 mg in cycle 2 and 20 mg in cycle 3+). The patient achieved a sustained partial response with mSWAT score decreasing to less than 20 beginning in cycle 4. (Fig 2, *A*-*C*).

**Fig 2:**
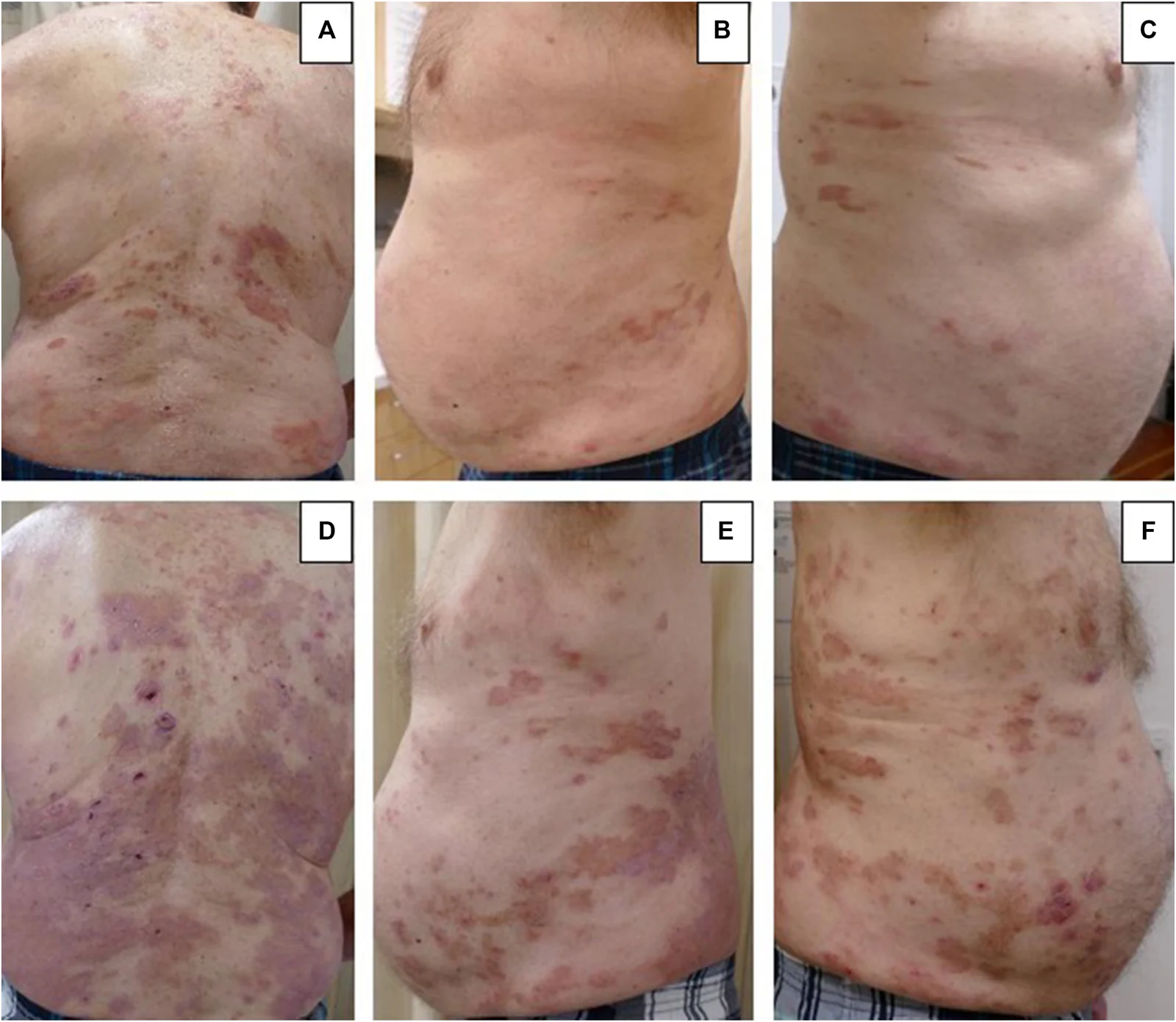
Clinical features of residual mycosis fungoides presenting with patches and plaques, 1 month prior onset of erythema multiforme-like lesions **(A-C)**. Erythema multiforme-like eruption presenting with large patches with focal erosions, partially confluent with underlying mycosis fungoides lesions on posterior trunk and left flank **(D** and **E)**. Right flank demonstrating a predominance of smaller, discrete patches with central erosions arising on previously uninvolved skin **(F)**.

However, on day 1 of cycle 22, the patient presented with a new eruption characterized by erythematous to dark red patches with focal erosions (Fig 2, *D*-*F*), primarily confluent over the back, buttocks, and posterior thighs—areas of previously dense MF involvement—with additional scattered lesions on the abdomen, arms, and anterior legs and previously unaffected skin. Notably, mucosal surfaces were spared. The patient reported that the eruption began 1 week after discontinuing triamcinolone accompanied by mild pruritus. There were no recent infections, new medications, or dietary changes.

Histopathologic evaluation of a new lesion demonstrated interface dermatitis with subepidermal vesiculation and features suggestive of leukocytoclastic vasculitis, consistent with an erythema multiforme (EM)-like drug eruption (Fig 3, *A* and *B*). There was no histopathological evidence of MF. Immunohistochemistry revealed a CD3+ T-cell infiltrate with normal CD4:CD8 ratio. Lymphocytes were focally positive for PD1, and approximately 30% of infiltrating histiocytes/macrophages expressed PD-L1 (Fig 3, *C*-*F*). The skin specimen was negative for herpes simplex virus (HSV)-1/2. Laboratory investigations including CBC, CMP, BP160 and BP230 serology, and direct immunofluorescence were unremarkable.

**Fig 3:**
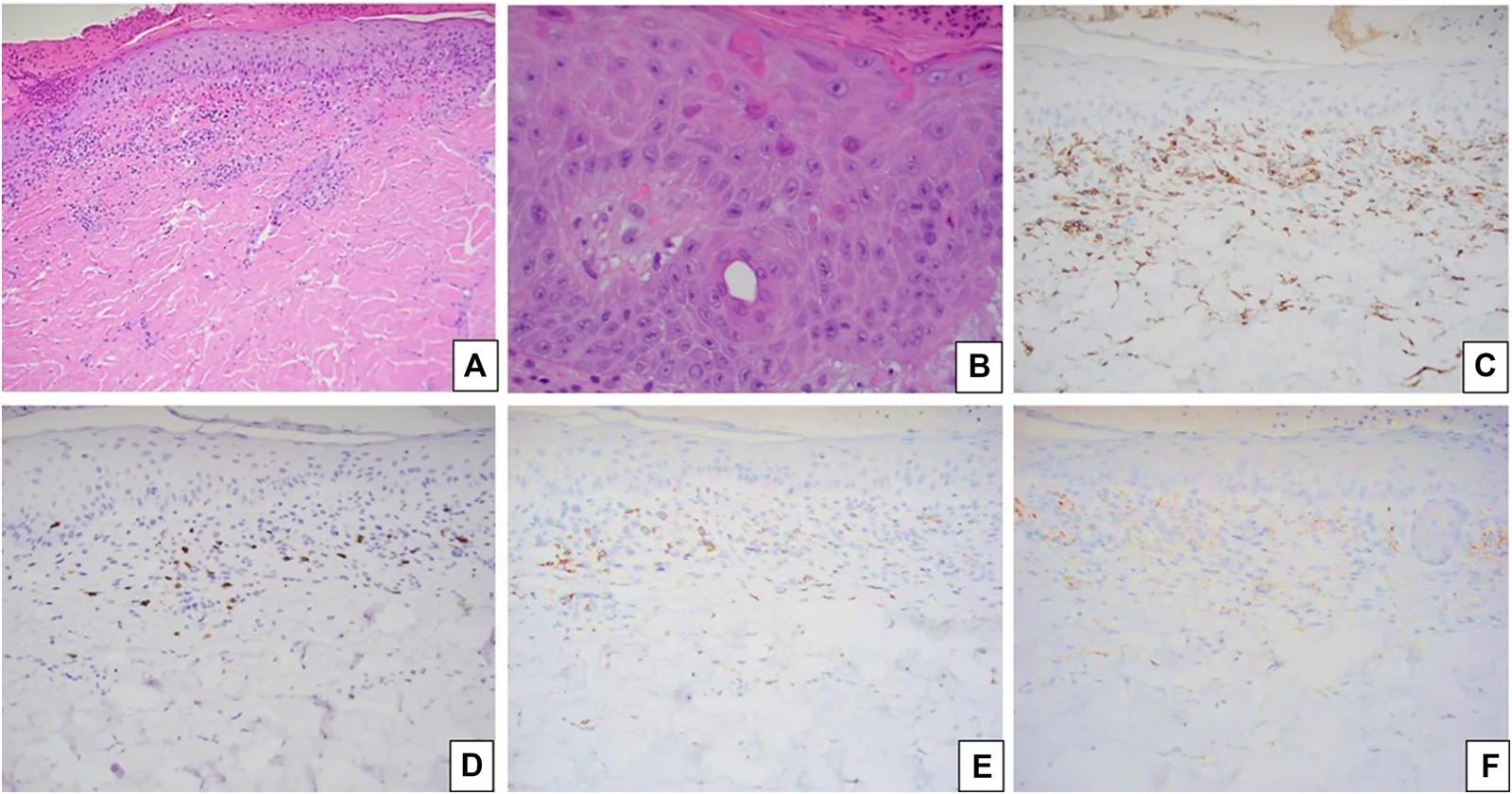
Histopathological and immunohistochemical features of erythema multiforme-like eruption demonstrate an interface dermatitis characterized by vacuolar degeneration of basaloid keratinocytes, and scattered necrotic keratinocytes extending to the acrosyringium, as well as extravasation of erythrocytes and neutrophils centered around small dermal blood vessels suggestive of leukocytoclastic vasculitis **(A** and **B)** [Hematoxylin-Eosin, original magnification ×100 and ×400]. By immunohistochemistry, the T cells show expression of CD4 **(C)**, scattered positivity for CD8 **(D)** and PD1 **(E)**. PD-L1 expression is observed in approximately 30% of infiltrating histiocytes and macrophages **(F)** [original magnification: ×200 **(C-F)**].

The eruption was graded as CTCAE grade 2 EM-like drug reaction. Both durvalumab and lenalidomide were discontinued. The patient was treated with triamcinolone cream twice daily, resulting in resolution of the EM lesions within 2-3 weeks. Subsequently, the patient experienced progression of CTCL lesions, though repeat biopsy showed no evidence of LCT, and was transitioned to an alternative systemic therapy.

## Discussion

Overall, the toxicity profile is more favorable with anti-PD-L1 compared with anti-PD1 therapy such as pembrolizumab that prevent interaction of PD1 with both PD-L1 and PD-L2.^6^ Previous irAEs reported in our phase 1/2 clinical trial of durvalumab with/without lenalidomide for refractory/recurrent CTCL were mostly mild to moderate in intensity and included tumor flare, fatigue, diarrhea, anemia, thrombocytopenia and autoimmune thyroiditis.^2^^,^^3^ Durvalumab-associated EM is a rare occurrence, with a reported odds ratio of 2.31.^8^ The late onset in this case suggests durvalumab as the primary driver, although lenalidomide’s contribution cannot be excluded. This timing aligns with delayed ICI-related events, whereas lenalidomide-induced EM typically occurs within the first month of therapy.^6^^,^^9^

By blocking PD-L1, durvalumab disrupts the inhibitory signaling essential for maintaining peripheral tolerance, thereby promoting T-cell activation against both tumor and cutaneous self-antigens. The PD1/PD-L1 axis serves as a critical checkpoint in skin homeostasis; its inhibition releases a brake on autoreactive CD4+ and CD8+ T cells, facilitating their infiltration into the skin and dermo-epidermal junction. This triggers a cytotoxic immune response against keratinocytes, which is a hallmark of EM-like eruptions. Notably, the high density of PD-L1-expressing macrophages and histiocytes within the CTCL microenvironment may have amplified local immune activation and subsequent tissue damage, potentially explaining the predilection of the eruption for areas with previous high tumor burden.

The absence of mucosal involvement and lack of bullae justified management with topical steroids alone. While current literature suggests that ICI rechallenge is often feasible, irAEs may reoccur with similar patterns upon re-exposure.^10^ Consequently, a rigorous benefit-risk assessment and vigilant clinical monitoring are mandatory if the inciting agent is resumed.

In conclusion, EM-like eruptions associated with durvalumab and lenalidomide may exhibit a delayed onset and remain flat without evolving into targetoid lesions, and generally are manageable with standard interventions. The concentration of EM-like eruptions around CTCL lesions suggests that the tumor microenvironment may serve as a site of heightened immune activation. Accurate differentiation between cutaneous irAE and CTCL progression remains paramount to ensuring appropriate management of these patients.

## Conflicts of interest

Crisan, Quach, Zain, and Rosen: none. Querfeld: Advisory board/Steering committee for SLAM BioTherapeutics, Helsinn, Kyowa Kirin, and Citius Pharmaceuticals; research grants: Helsinn and Kyowa Kirin; clinical investigator: Kyowa Kirin, SIRPant, and BioInvent.
